# Evidence of motor injury due to damaged corticospinal tract following acute hemorrhage in the basal ganglia region

**DOI:** 10.1038/s41598-020-73305-8

**Published:** 2020-10-01

**Authors:** Jing Li, Xue Hu Wei, Yong Kang Liu, Ling Shan Chen, Zheng Qiu Zhu, Si Yuan Hou, Xiao Kun Fang, Zhong Qiu Wang

**Affiliations:** 1 Department of Radiology, Affiliated Hospital of Nanjing University of Chinese Medicine, Jiangsu Province Hospital of Chinese Medicine, Nanjing, 210029 China; 2 Max Planck Institute for Human Cognitive and Brain Sciences, 04103 Leipzig, Germany; 3 Department of Ultrasound, Affiliated Hospital of Nanjing University of Chinese Medicine, Jiangsu Province Hospital of Chinese Medicine, Nanjing, 210029 China; 4 Department of Acupuncture and Rehabilitation, Affiliated Hospital of Nanjing University of Chinese Medicine, Jiangsu Province Hospital of Chinese Medicine, Nanjing, 210029 China

**Keywords:** Stroke, Basal ganglia

## Abstract

The integrity of the corticospinal tract (CST) is significantly affected following basal ganglia haemorrhage. We aimed to assess the local features of CST and to effectively predict motor function by diffusion characteristics of CST in patients with motor injury following acute haemorrhage in the acute basal ganglia region. We recruited 37 patients with paresis of the lateral limbs caused by acute basal ganglia haemorrhage. Based on the automated fiber quantification method to track CST, assessed the character of each CST segment between the affected and contralateral sides, and correlated these with the Fugl–Meyer (FM) and Barthel Index (BI) scores at 6 months after onset. The fractional anisotropy (FA) values of the injured side of CST showed a significantly lower FA than the contralateral side along the tract profiles (p < 0.05, corrections for multiple comparisons). The FA values of each site at the internal capsule, closed corona radiata were positively correlated with the FM and BI score at 6 months after onset (p < 0.001, respectively). Our findings assessed the character of CST vividly in detail and dementated the primary sites of CST can predict the long-term outcome of motor function. This study may facilitate future clinical and cognitive studies of acute haemorrhage.

## Introduction

The basal ganglia region is involved in many neuronal pathways having emotional, motivational, associative, and cognitive functions, forming a major center in the complex extra pyramidal motor system^1^. Haemorrhage in the region of the basal ganglia is a common cause of experiencing residual motor weakness or long-term disability^2,3^, typically, neural injury by intracerebral haemorrhage (ICH) can result from a mass effect of hematoma and secondary injury of the peri-hematomal white matter due to increased intracranial pressure, decreased perfusion, and brain edema^4–7^, such damage might result in abnormal diffusion of water. likely because ICH squeezes or destroys the motor function neural tract, particularly the corticospinal tract (CST) which is the most important neural tract for motor control in the human brain^5,6,8^. Many previous studies have attempted to estimate the state of CST in patients with stroke^6,9,10^ and reported that the CST can predict motor impairment to achieve good motor function in stroke patients^3,11^. Therefore, as per Lee’s study, preventing or minimizing CST injury could be crucial for initial treatment in patients with putaminal haemorrhage^6^. Tract-based spatial statistics (TBSS) was used to estimate the affection of CSTs’ skeletone^12,13^. And most previous studies are based on different methods to evaluate injury of lateral CST in patients with intracerebral haemorrhage^6,9,14–16^ by manually placing ROI, which may lead to inadequate repeatability and accuracy.

Besides, reduced diffusion properties of CST at the different position may be caused by different reasons, reduced FA at the cerebral peduncle is distal to the putamen and the thalamus is supposed to be attributed to Wallerian degeneration^17^, the decreased FA data at the internal capsule might be interfered with by hemosiderin^16^. Moreover, different locations along the corticospinal tract (CST) with different prediction index to the motor outcome^16,18^. To study the influence of this different location along the corticospinal tract (CST) and association to motor outcome, the most previous studies tried to draw different ROI and estimated the ROI’s diffusion character in the middle corona radiata, the posterior limb of the internal capsule, and the cerebral peduncle along the CST pathway^18–21^. However, few studies reported the detailed characteristics of each successive segment along the CST. In this study, we investigated the diffusion characteristics of CST in each small segment, based on the diffusion tensor imaging (DTI) method (Automated Fibre Quantification, AFQ) in 37 patients with acute basal ganglia haemorrhage, and evaluated the correlation between CST impairment and motor function score. Demonstrating the whole CST with detailed segments could be helpful to evaluate motor disability caused by acute basal ganglia haemorrhage and to improve the motor resilience during clinic treatment.

## Results

**Figure 1:**
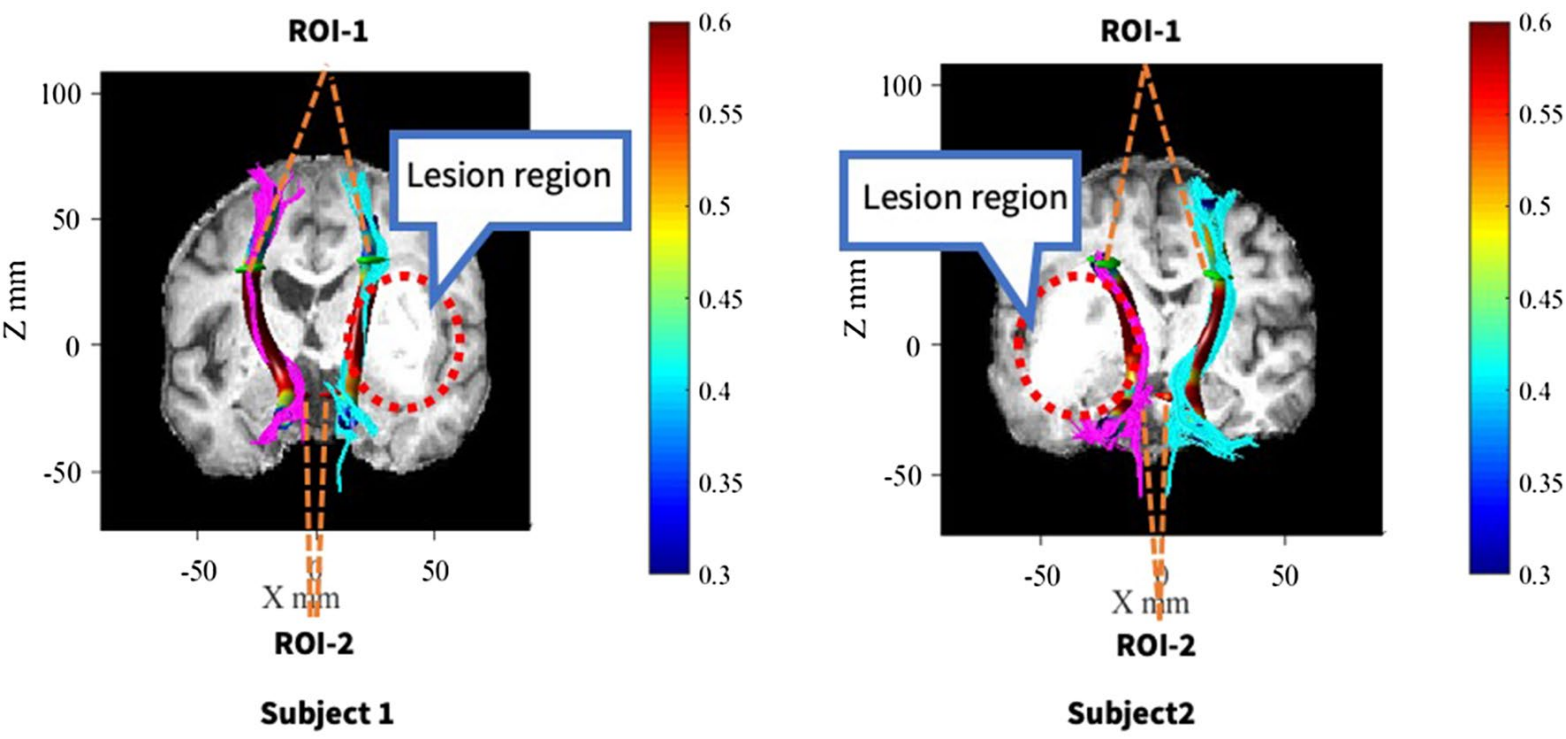
Diffusion tensor tractography of two subjects with cerebral haemorrhage in the putamen. Subject 1: A 45-year-old man with cerebral haemorrhage in right hemisphere. Subject 2: A 52-year-old man with cerebral haemorrhage in left hemisphere. Fuchsia lines stand for CST fibers on the left hemisphere, and sea green lines stand for CST fibers on the right hemisphere. The color tubes around the fibers represent the FA value along to the fiber bundle. The color bar corresponds to the color of the tubes and represents the range of FA values.

**Figure 2:**
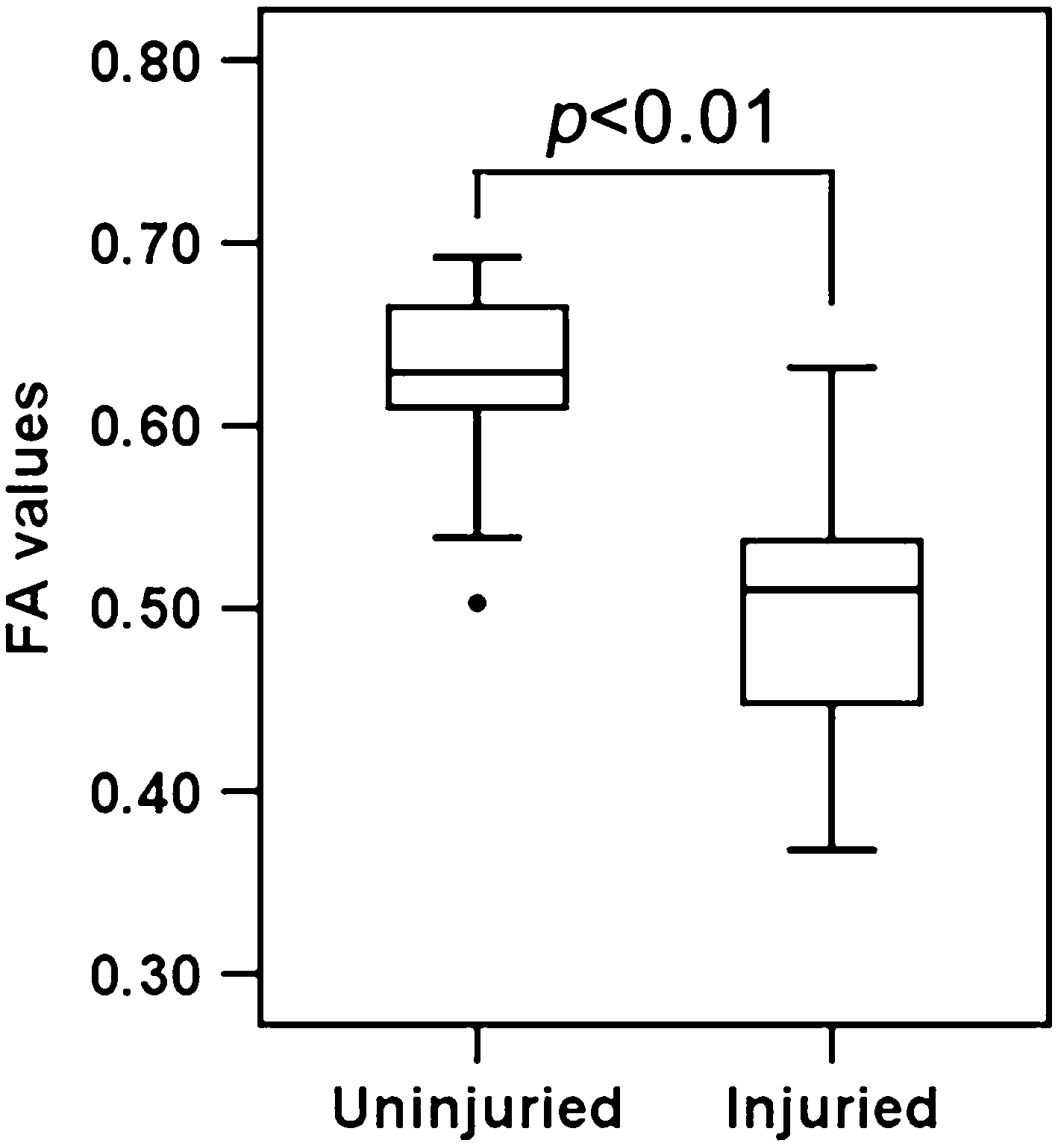
Mean FA and standard deviation between the injured and uninjured sides.

**Figure 3:**
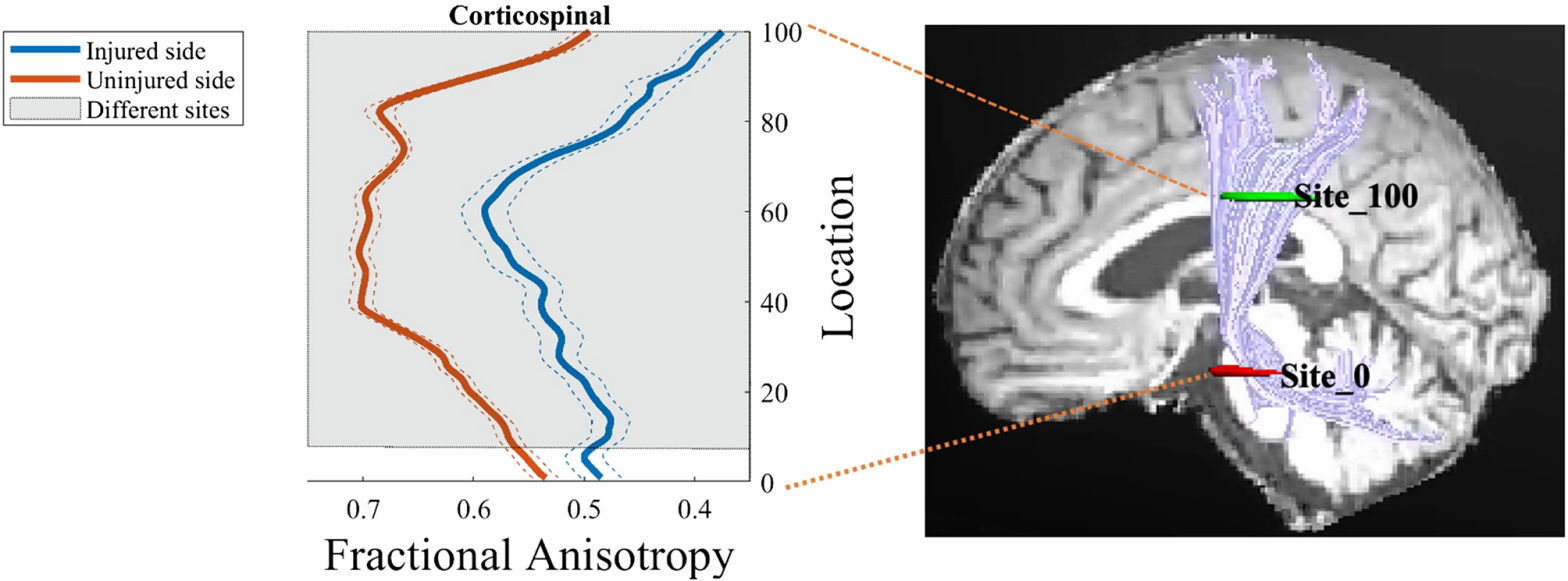
Point-wise comparisons of FA value along the CST between the injured and uninjured sides. The horizontal scale represents 100 equidistant sites along the central portion of the tract. The blue solid line refers to the mean FA value of the injured side, while the orange solid line refers to the mean FA value of the uninjured side. The dashed lines denote the SD values. Shaded areas represent sites with significant statistical difference in the FA value between the injured and uninjured sides (p < 0.05, FDR corrected).

Compared with the CST of the uninjured side, that on the injured side showed sparse and varied degrees of deformation in Fig. 1. First, two-sample t-tests performed on the mean FA value of entire CST between the injured and uninjured side showed that the mean FA of the affected CST was significantly lower than that of the unaffected side (p < 0.01) as in Fig. 2. To demonstrate the main affected locations, we calculated the FA in each site (100 nodes) along the CST, then the t-test was performed on each site’s FA to distinguish between the injured and uninjured sides. The results indicated that except measurement sites closed to the cerebral peduncle, the FA of each site located from the bottom (8th point) to top (100th point) along the CST of the injured side was significantly lower (p < 0.05, FDR corrected) than the uninjured side (Fig. 3, were marked with a shadow area). It will be helpful to locate the major affected positions of the CST.

**Figure 4:**
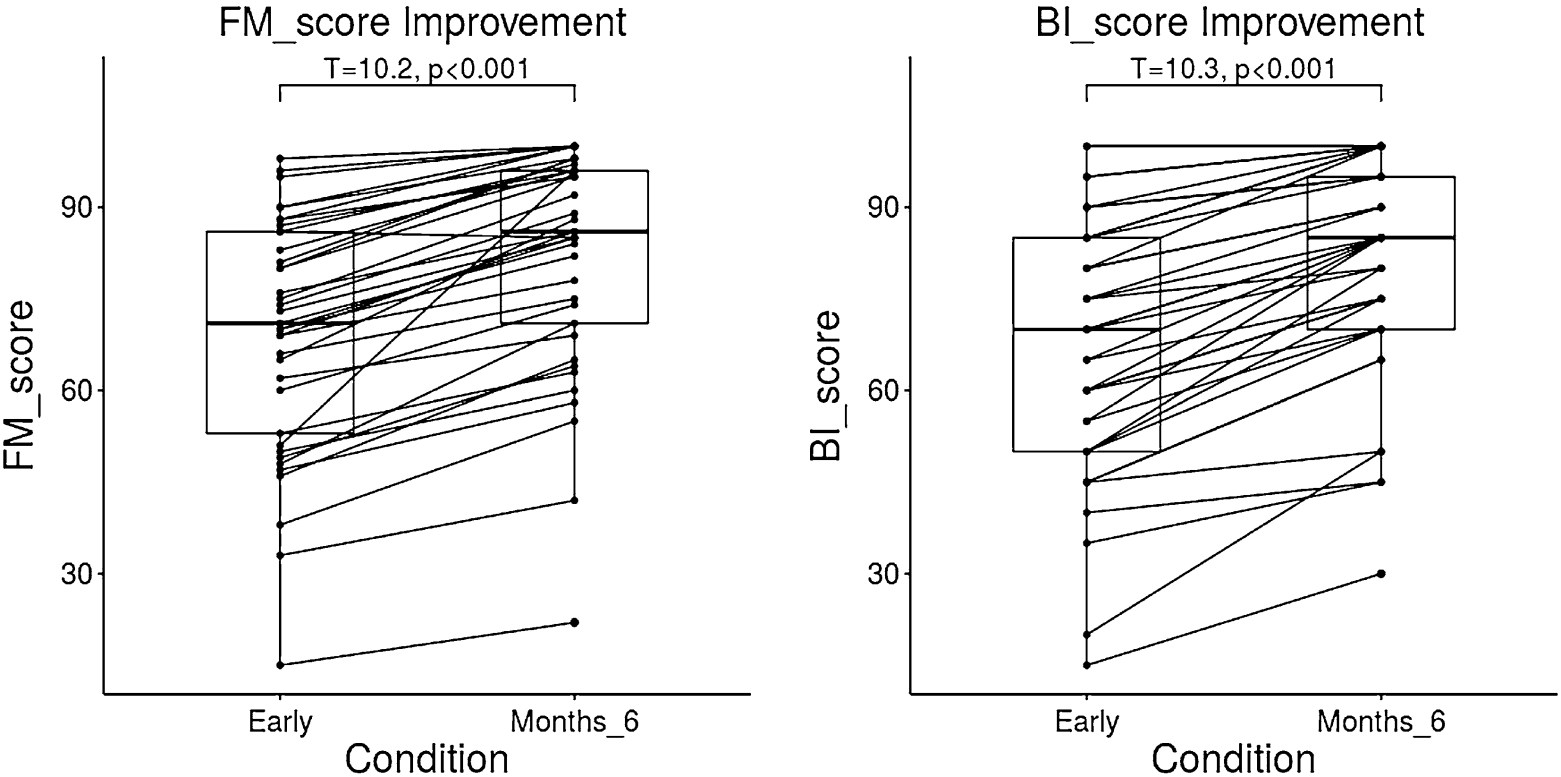
Comparison of the motor outcomes between the time of admission and 6 months after onset.

**Figure 5:**
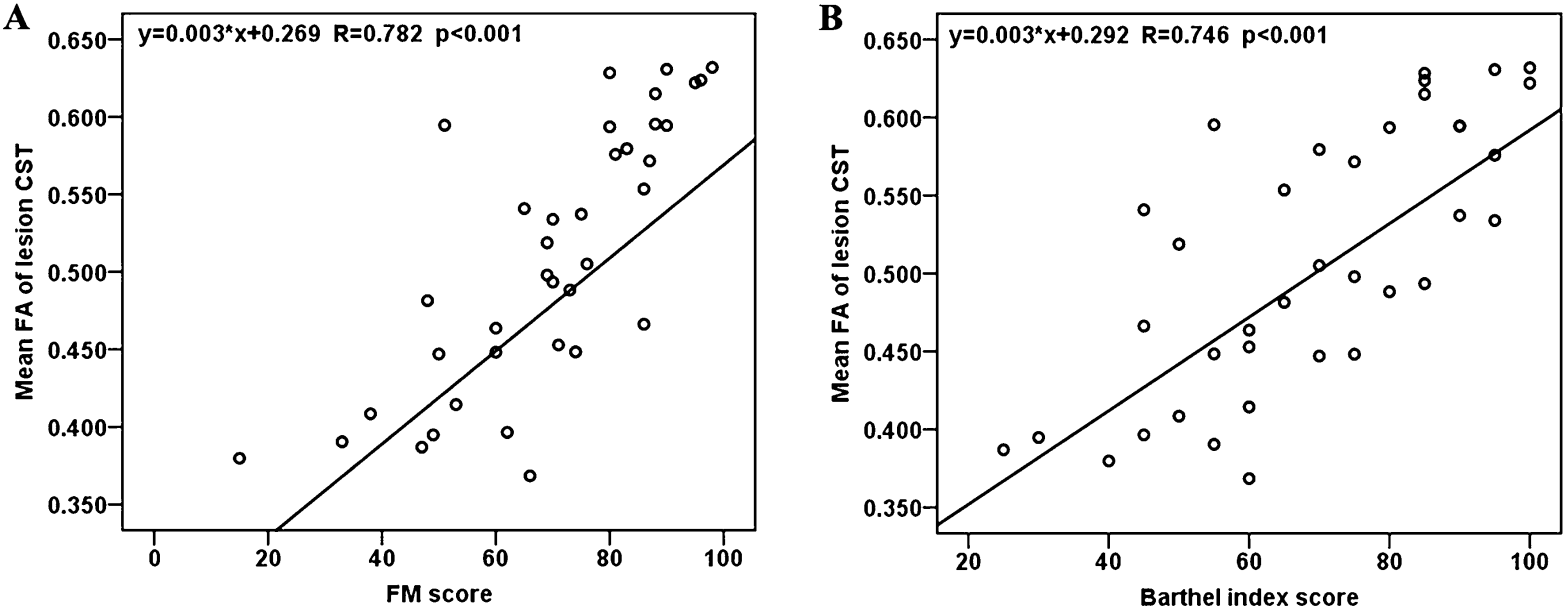
The mean FA of the lesion CST showed significant correlation with FM score (r = 0.782, p < 0.001) and BI score (r = 0.746,  p < 0.001) during the 6-month follow-up evaluation.

**Figure 6:**
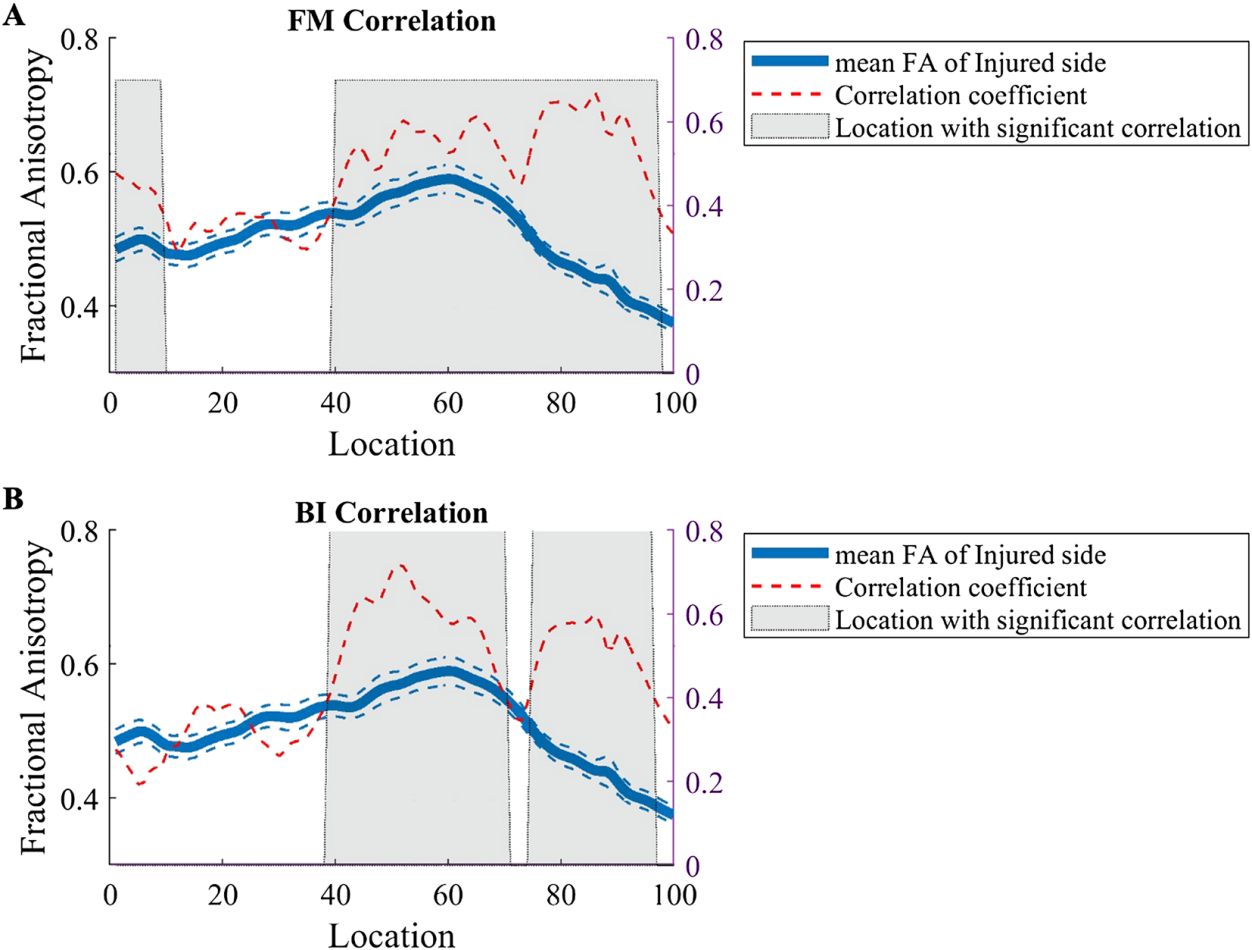
Point-wise correlation of FA value along the CST with FM score and BI score during the 6-month follow-up evaluation. The blue solid line refers to the mean FA value of the injured side. The blue dashed lines denote the SD values. The red dashed line represents the correlation index at each site along the CST. Shaded areas represent sites with significant statistical correlation between FA and FM score and BI score, respectively (p < 0.05, FDR corrected).

Compared with the motor outcomes at the time of admission, the function was clinical improvement at 6 months after onset, with a significant increase of the FM scores (paired t-test, T = 10.173, p < 0.001), BI scores (paired t-test, T = 10.299, p < 0.001) as in Fig. 4. A regression analysis conducted to assess the change of CST will help to evaluate the motor disability due to acute basal ganglia haemorrhage and help to predict clinical long-term outcome of motor function. The mean FA of the affected CST segments showed a significant correlation with the FM score (r = 0.782, p < 0.001) as Fig. 5A, and BI score (r = 0.746,  p< 0.001) at the 6-month follow-up evaluation Fig. 5B. Moreover, the same regression analysis was performed at each site along the CST to test the primary location where domain the predicational index for the long-term motor ability. A short segment of CST (1st-10th point ) close to cerebral peduncle with significant corelation between FA and 6-month’s FM score(r = 0.38 ~ 0.60, p < 0.05, FDR corrected) . The main sites of CST with a significant correlation between FA and 6-month’s FM score was found at the region of the internal capsule/corona radiata (38th-97th point, r = 0.38–0.70, p < 0.05, FDR corrected) as in Fig. 6A. The main sites of CST with a significant correlation between FA and 6-month’s BI score was found at the region close to internal capsule/corona radiata (38th–97th point, r = 0.38–0.72,  p< 0.05, FDR corrected) as in Fig. 6B.

## Discussion

In this study, we attempted to investigate the differences in CST characteristics between the injured and uninjured sides of patients following an ICH. Our results can be summarized as follows: (1) Intracerebral haemorrhage significantly affected the CST diffusion characteristic. (2) On follow-up brain MRI, CST diffusion characteristics were significantly associated with motor ability score, meaning that the robustness of CST may affect the resilience of motor ability post-ICH.

Damage and alteration of CST characteristics have been reported as a major aspect of ICH, and the associated motor weakness seems to correlate with CST impairment^2,3,5,6^. Our study showed that the diffusion characteristics of the injured-side CST had a lower mean FA than the uninjured side. To determine the different location of CST impairment, we segmented the CST into 100 equal parts and compared the differences in each subdivision point along the CST using a previously described method in a study on stroke patients^22^. We found that the segments of the injured-side CST that were close to the cerebral peduncle and posterior inner capsule showed a significant decrease in mean FA. The decrease in FA in middle, and top segments (21–100th point) along the CST where close to the cerebral peduncle and posterior inner capsule is mostly caused by compression, displacement, destruction, or cutting of the haematoma^18^. Such damage to the whiter matter by acute haemorrhage in the basal ganglia region will change the integrity and spatial direction of axonal membranes and myelin sheath of the fibrous bundle, thereby affecting the water molecular diffusion barrier to increase the free diffusion of water molecules and decrease the diffusion anisotropy of white matter in lesions, ultimately leading to a decrease in the FA value^23^. Studies have also shown that the FA value near the hematoma area is affected by the magnetic susceptibility of hemosiderin deposition or mixed vasogenic and cytotoxic oedema^16,24^. The FA value in the bottom segments (8–20th point) along the CST of the injured side was significantly lower than the uninjured side, such segments close to cerebral peduncle that did not close the lesion position also likely attributed to the significantly lower FA value in our study. This may also be because of the wallerian degeneration of the damaged brain white matter^17,25,26^.

The FM and BI scores allowed measurement of motor function reliability at the motor behaviour level^27^. The CST characteristics may be able to reliably reflect behaviour restoration. In this study, we investigated the recovery course of the motor ability at 6 months in patients with ICH, we found that FM and BI scores with significantly increased at 6 months compared with early stage, demonstrated an improvement of motor outcomes. In addition, our study results showed that the mean FA value of the affected-side CST with different segments was significantly positively correlated with the follow-up FM and BI scores at 6 months. The correlation between CST and behaviour reliability in our study is consistent with previous DTI-based studies^10,28,29^. This result indicated that the degree of CST involvement in stroke was directly related to stroke severity and functional recovery and suggested that long-term recovery was related to the integrity and directional preservation of white matter fibres. In essence, our study demonstrated robust evidence that diffusion tensor tractography of the CST can be used early to evaluate and predict the patient’s motor function prognosis and independent living ability^3,15,30^. In addition, in the current study, the determination of the DTI data from different locations along the corticospinal tract (CST) pathway and association to the motor outcome of flow-up at 6 months reveals that the corona radiata and the posterior limb of the internal capsule might be the primary locations for predicting the long-term motor recovery. This finding is consistent with previous studies^16,20^. But most previous studies have used only several pointed ROIs included corona radiata, perihematomal edema area, cerebral peduncle, and pons, as the ROI for FA measurements to predict motor outcomes. Our study is unique in that we assessed 100 sites along the CST, to measure DTI indices and association to the motor outcome to find the primary locations of CST affected by ICH, as well as the primary locations where contribute for the motor recover ability prediction. Our study was based on the AFQ method for tractography and detailed analysis of the CST state, which can demonstrate that three-dimensional axonography is constructed using the diffusion-weighted image in the early stages of stroke (within 15 days after onset) but not only estimate in the skeleton of wither matter as the previous studies^12^. This study can provide valuable information for clinical practice, guide clinical work better, and ensure effective intervention measures.

## Materials and methods

### Subjects

This study included 37 patients with haemorrhage at the acute basal ganglia region from a dataset was recruited from February 2016 to April 2018. The patient inclusion criteria were as follows: (1) the course of the disease was within 15 days; (2) the haemorrhage was located in the region of the basal ganglia (putamen or thalamus); (3) no history of stroke, brain injury, or other neurological disorders; (4) medicine treatment method, no surgery; and (5) with more or less paresis of the lateral limbs at the time of admission. All patients underwent routine medical treatment and rehabilitation therapy within 6 months. The mean age of the patients (27 males, 10 females) was 54.7 ± 10.1 years (range: 30–75 years). 22 and 15 patients had ICH in the left and right hemispheres, respectively. 11 patients had lesions in the putamen; 20 patients had lesions within the thalamus; and 6 patients had lesions in the putamen extending up to the thalamus. The median time interval from onset of ICH to the MRI study was 9 ± 3 days. The study protocol was approved by the ethics committee of the Affiliated Hospital of Nanjing University of Chinese Medicine, and informed consent was obtained from all patients. And all the experiments and the methods were performed in accordance with the approved guidelines and regulations.

### MRI data acquisition

All patients underwent MRI scans within 14 days of admission. Scanning of the dataset was performed on a 3 T MRI scanner (Siemens). For each participant, a high-resolution structural T1 weighted volume of the whole brain was acquired (TR/TE = 1900/2.99 ms; slice thickness = 0.9 mm; field of view = 201 mm × 230 mm; matrix size = 220 × 256; 160 slices with no gap; voxel sizes = 0.9 × 0.9 × 0.9 mm; and flip angle = 9°). The DTI data were acquired using a spin-echo, single-shot, echo-planar imaging sequence (TR/TE = 10,000/95 ms; slice thickness = 2 mm; field of view = 256 mm × 256 mm; matrix size = 128 × 128; voxel sizes = 2 × 2 × 2 mm; 70 slices with no gap; b = 1000 s/mm^2^; and number of gradient directions = 30. One non-diffusion weighted image was acquired before diffusion-weighted measurements.

### Clinical evaluation of motor

The upper and lower extremity sections of the Fugl–Meyer motor function assessment scale (FM motor scale) were primarily used to measure impairment of motor ability. The FM scale has excellent reliability and high predictive validity for outcome^31^. In addition, we used the Barthel Index (BI) that is most commonly used to measure disability that affects the basic activities of daily living^32^. The above scores were evaluated at the time of admission and 6 months by experienced therapists after ICH onset, scores of each subject as showed in Supplementary Table 1. Such two motor function assessment scales were used to assess different degrees of motor deficit at the time of admission and motor improvement ability in 6 months’ treatment, respectively.

### Imaging data pre-processing

The pre-processing of DTI data was performed as follows: (1) Diffusion images were corrected for eddy current distortions and subject head motion with the FMRIB’s Diffusion Toolbox (FDT); (2) Non-brain tissue was deleted with Brain Extraction Tool (BET); and (3), Diffusion tensor construction of individual fractional anisotropy (FA) maps and S0 (raw T2 signal with no diffusion weighting) was locally fitted using DTIFIT from FMRIB’s Diffusion Toolbox. All pre-processing was carried out using the FMRIB Software Library (FSL) (www.fmrib.ox.ac.uk/fsl).

### Automated fibre quantification

The quantification of white-matter fibre bundles was performed by the AFQ software (https://github.com/jyeatman/AFQ). First, we used the dtiMakeDt6 function from (https://github.com/vistalab/vistasoft/tree/master/mrDiffusion) to convert the S0 image (raw T2 signal with no diffusion weighting, which was estimated by FSL) in the previous step in mrDiffusion format to create a dt6.mat file that was used for the next process. Then, quantification of white-matter fibres was performed as described previously^33^. The following six steps were implemented by AFQ software: (1) Whole brain tractography was initiated from each white matter voxel with FA > 0.3, individual streamline integration is terminated using two standard criteria: tracking is halted if (1) the FA estimated at the current position is below 0.2 and (2) the minimum angle between the last path segment and next step direction is greater than 30°. (2) Two-way point regions of interest (ROIs) were used to segment the fibres referring to previous studies^34^. (3) Each candidate fibre was then scored based on its similarity to a standard fibre tract probability map, and fibres with high probability scores were retained. (4) Fibre tracts were represented as a 3-dimensional Gaussian distribution, and outlier fibres that deviated substantially from the mean tract position were removed. (5) The fibre group to the central portion that spanned the two defining ROIs was clipped. (6) The fibre group core was calculated by resampling each fibre into 100 equidistant segments, and the FA of each segment as the main estimation parameter of the fiber. Diffusion measurements were calculated at each segment by considering a weighted average of the FA measurements of each individual fibre’s diffusion properties at these equidistant segments. FA of each segment along the fiber stand for the diffusion character of each site of the fiber.

### Statistical analysis

CST fiber tract was done by AFQ, then each CST was resampled into 100 equidistant segments, the FA of each segment stand for the FA of the different site along the CST from bottom to up. Firstly, we calculated the mean FA of the entitle CST by averaged the FA valuate at each site (100 segment in total ) , and independent two-sample t-tests were performed on the mean FA of injured and uninjured CST to determine the extent of CST affection due to acute haemorrhage in the region of basal ganglia. Then the independent two-sample t-test was also used to test the difference of FA in each site (100 nodes in total) of the CST between the injured and uninjured side to locate the injury positions. In order to access the improvement of motor function outcome, difference of FM score and BI score between the early stage and 6 months after onset were investigated using paired t-tests. Pearson’s correlation test was used to assess the correlation between the mean FA value of the affected CST segments and the FM score to estimate the relation between CST and restore ability of motor function; a separate analysis was performed for the BI score; regression the to exclude the affection for gender and age. The same Pearson’s correlation test was used at each site (100 sites) along CST to test the correlation between each local site’s FA and FM, BI score respectively.The threshold at a level of uncorrected p < 0.05 of statistical parametric maps was considered, and at a multiple comparison level, corrected p < 0.05 was considered using the false discovery rate (FDR).

### Limitations

There are limitations to our study. Due to methodological limitation, our study does not allow further detection of different injured position of tracts that may be due to the lesion result in dying back of axons, death of their layer 5 pyramidal neurons, wallerian degeneration or potentially toxic effects of heme products. In addition, hematoma volume was missing in our study, these important aspects will be included in our next study. We focused mainly on the feature of CST, and try to use early CST’s feature for early predictions of long-term outcome of motor function, it may be helpful for rehabilitation planning of ICH.

## Conclusions

Our study sheds slights on how the CST is affected by intracerebral haemorrhage in the early stages. In addition, the diffusion characteristics of CST in the early stage are likely effective predictors of the restoration ability of motor function in patients with haemorrhagic stroke. More importantly, our study detailed denominated the main locations along CST where effectively predicting the recovery ability of motor outcome.

## Supplementary information

Supplementary Information.
