# Supplementary material for: Evidence of motor injury due to damaged corticospinal tract following acute hemorrhage in the basal ganglia region
Source: Sci Rep. 2020 Oct 1;10:16346. doi: 10.1038/s41598-020-73305-8 (PMC7530683; doi:10.1038/s41598-020-73305-8)
Supplement: Supplementary file 1 — Supplementary Information. [file 41598_2020_73305_MOESM1_ESM.docx]

**Supplementary information**

**Evidence of motor injury due to damaged corticospinal tract following acute hemorrhage in the basal ganglia region**

Jing Li^1^, Xue Hu Wei^2^, Yong Kang Liu^1^, Ling Shan Chen^1^, Zheng Qiu Zhu^3^,Si Yuan Hou^4^, Xiao Kun Fang^1^& **Zhong Qiu Wang^1^**

^1^Department of Radiology, Affiliated Hospital of Nanjing University of Chinese Medicine, Jiangsu Province Hospital of Chinese Medicine，Nanjing 210029, China

^2^Max Planck Institute for Human Cognitive and Brain Sciences,04103, Leipzig, Germany

^3^Department of Ultrasound, Affiliated Hospital of Nanjing University of Chinese Medicine, Jiangsu Province Hospital of Chinese Medicine，Nanjing 210029, China

^4^Department of acupuncture and rehabilitation ,Affiliated Hospital of Nanjing University of Chinese Medicine, Jiangsu Province Hospital of Chinese Medicine，Nanjing 210029, China

﻿Correspondence and requests for materials should be addressed to Zhong Qiu Wang (email: [zhqwang001@126.com](mailto:zhqwang001@126.com))

**Table 1 motor functional outcomes**

|  | **FM score** | | **Barthel score** | |
| --- | --- | --- | --- | --- |
| **﻿ Patient** | **Admission** | **Six months** | **Admission** | **Six months** |
| patient 1 | 65 | 88 | 50 | 80 |
| patient 2 | 15 | 22 | 40 | 45 |
| patient 3 | 95 | 100 | 100 | 100 |
| patient 4 | 50 | 60 | 70 | 80 |
| patient 5 | 33 | 42 | 45 | 50 |
| patient 6 | 76 | 86 | 65 | 85 |
| patient 7 | 46 | 65 | 35 | 45 |
| patient 8 | 80 | 98 | 80 | 100 |
| patient 9 | 53 | 63 | 60 | 75 |
| patient 10 | 49 | 64 | 20 | 50 |
| patient 11 | 70 | 85 | 70 | 85 |
| patient 12 | 75 | 92 | 90 | 95 |
| patient 13 | 60 | 74 | 75 | 80 |
| patient 14 | 74 | 89 | 55 | 70 |
| patient 15 | 98 | 100 | 100 | 100 |
| patient 16 | 86 | 85 | 45 | 65 |
| patient 17 | 81 | 98 | 95 | 100 |
| patient 18 | 62 | 69 | 45 | 65 |
| patient 19 | 73 | 85 | 80 | 90 |
| patient 20 | 86 | 97 | 60 | 85 |
| patient 21 | 87 | 96 | 75 | 90 |
| patient 22 | 69 | 86 | 50 | 75 |
| patient 23 | 88 | 95 | 55 | 85 |
| patient 24 | 47 | 58 | 15 | 30 |
| patient 25 | 66 | 75 | 60 | 75 |
| patient 26 | 51 | 96 | 90 | 100 |
| patient 27 | 88 | 100 | 85 | 100 |
| patient 28 | 90 | 100 | 95 | 100 |
| patient 29 | 83 | 96 | 70 | 85 |
| patient 30 | 70 | 82 | 85 | 95 |
| patient 31 | 71 | 84 | 60 | 70 |
| patient 32 | 80 | 95 | 80 | 90 |
| patient 33 | 69 | 78 | 75 | 85 |
| patient 34 | 96 | 100 | 85 | 100 |
| patient 35 | 90 | 98 | 90 | 95 |
| patient 36 | 48 | 71 | 65 | 75 |
| patient 37 | 38 | 55 | 50 | 70 |
